# Resolution of duodeno-jejunal dehiscence using a fully covered duodenal stent with trans-mesh biliary and pancreatic stenting

**DOI:** 10.1055/a-2976-9041

**Published:** 2026-10-28

**Authors:** Fabrizio Termite, Andrea Tringali, Ivo Boškoski, Vincenzo Perri, Claudio Lodoli, Fabio Pacelli, Cristiano Spada

**Affiliations:** 1 Digestive Endoscopy Unit 18654 Fondazione Policlinico Universitario Agostino Gemelli IRCCSRomeLazioItaly; 2 Department of Digestive Surgery 18654 Fondazione Policlinico Universitario Agostino Gemelli IRCCSRomeLazioItaly; 3 Department of Digestive Surgery 18654 Fondazione Policlinico Universitario Agostino Gemelli IRCCSRomeLazioItaly; 4 Digestive Endoscopy Unit 18654 Fondazione PoliambulanzaBresciaItaly

## Case description



**Video 1** Placement of a fully covered duodenal stent with trans-mesh biliary and pancreatic stenting for the treatment of a duodeno-jejunal anastomotic leak, followed by en bloc stent removal and endoscopic confirmation of complete healing. The Video link below:URI: .


Duodeno-jejunal anastomotic leakage is uncommon but potentially life-threatening postoperative complications. 1​ Surgical re-intervention is often challenging and associated with substantial morbidity. 1​

We present the case of a 64-year-old man who underwent multivisceral resection for retroperitoneal recurrence of testicular liposarcoma, with the creation of a duodeno-jejunal anastomosis distal to the papilla ( Video 1) . Two months after surgery, he developed fever and abdominal pain. Computed tomography (CT) demonstrated an anastomotic dehiscence associated with a large peri-anastomotic abscess.

A stepwise endoscopic approach was performed. First, a nasopancreatic drainage tube was placed. Subsequently, a 22-mm×15-cm fully covered self-expandable metallic stent was deployed across the duodenum to exclude the anastomotic defect and was secured proximally using a StentFix anchoring device. Through the mesh of the duodenal stent, selective cannulation of the biliary and pancreatic ducts was achieved. An 8-mm×8-cm fully covered biliary metal stent and a 6-mm×6-cm fully covered pancreatic metal stent were then placed, allowing the complete internal diversion of digestive secretions and removal of the external drains ( Fig. 1 ).

**Fig. 1:**
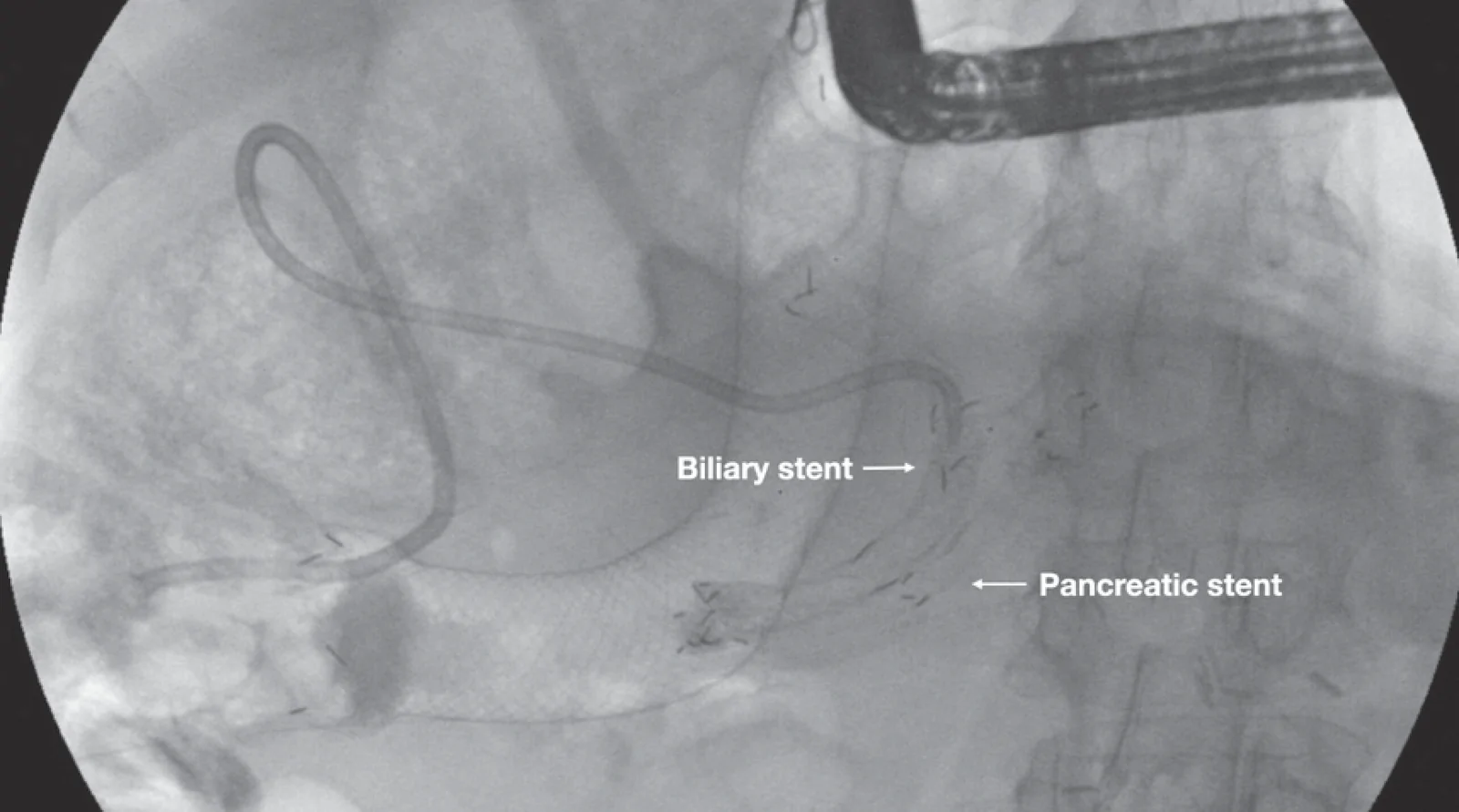
A fluoroscopic image showing the duodenal stent and the trans-mesh biliary and pancreatic stents.

The patient showed rapid clinical improvement and resumed a semi-liquid diet. Three months later, follow-up CT demonstrated the complete closure of the anastomotic leak with marked reduction of the associated fluid collection. Following dislodgement of the anchoring device, all three stents were removed en bloc ( Fig. 2 ). Endoscopic and fluoroscopic assessment confirmed complete healing of the anastomosis ( Fig. 3 ).

**Fig. 2:**
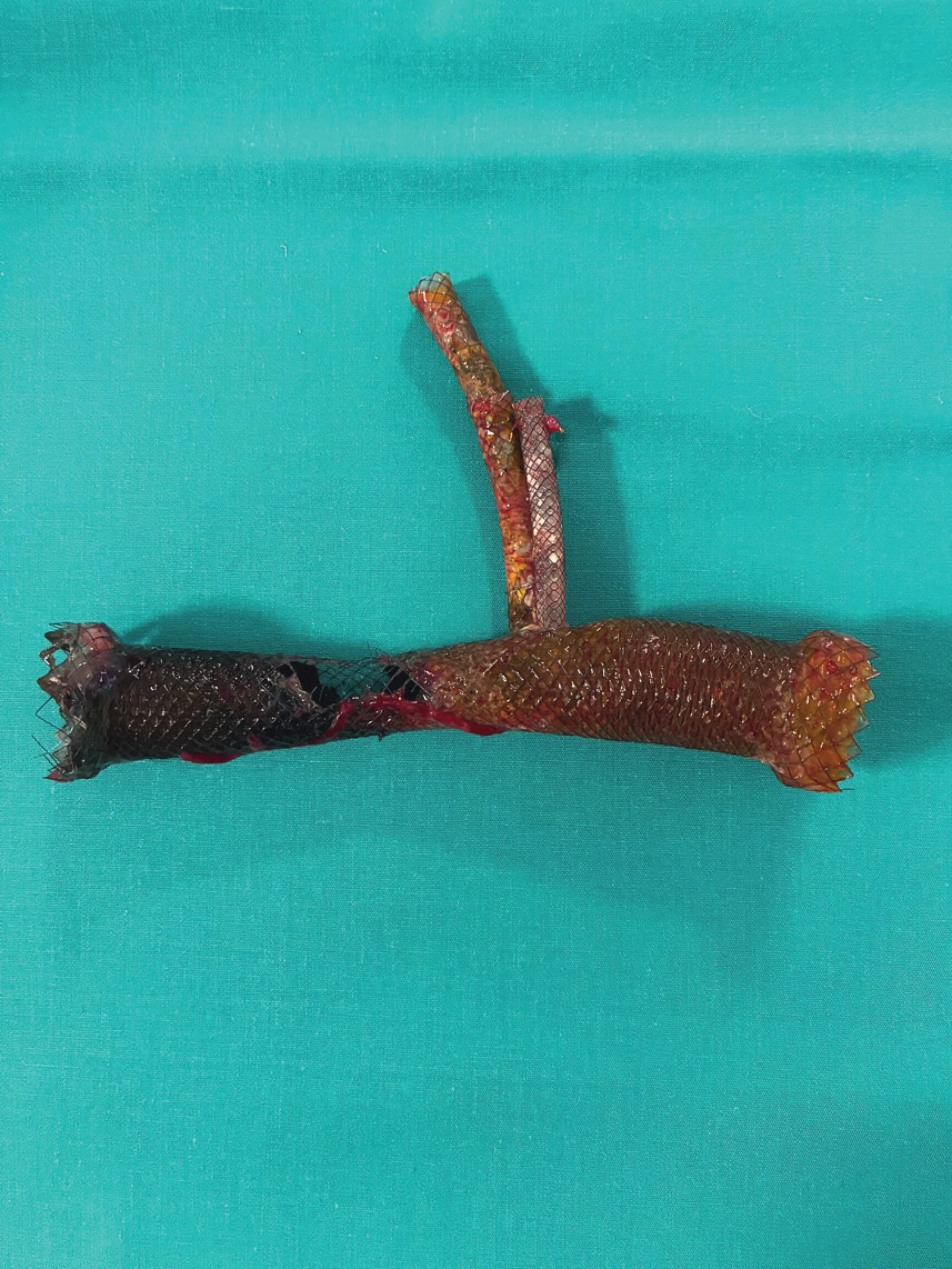
A photograph of the ‘en bloc’ removed stents, and a lateral view showing the duodenal stent with trans-mesh biliary and pancreatic stents.

**Fig. 3:**
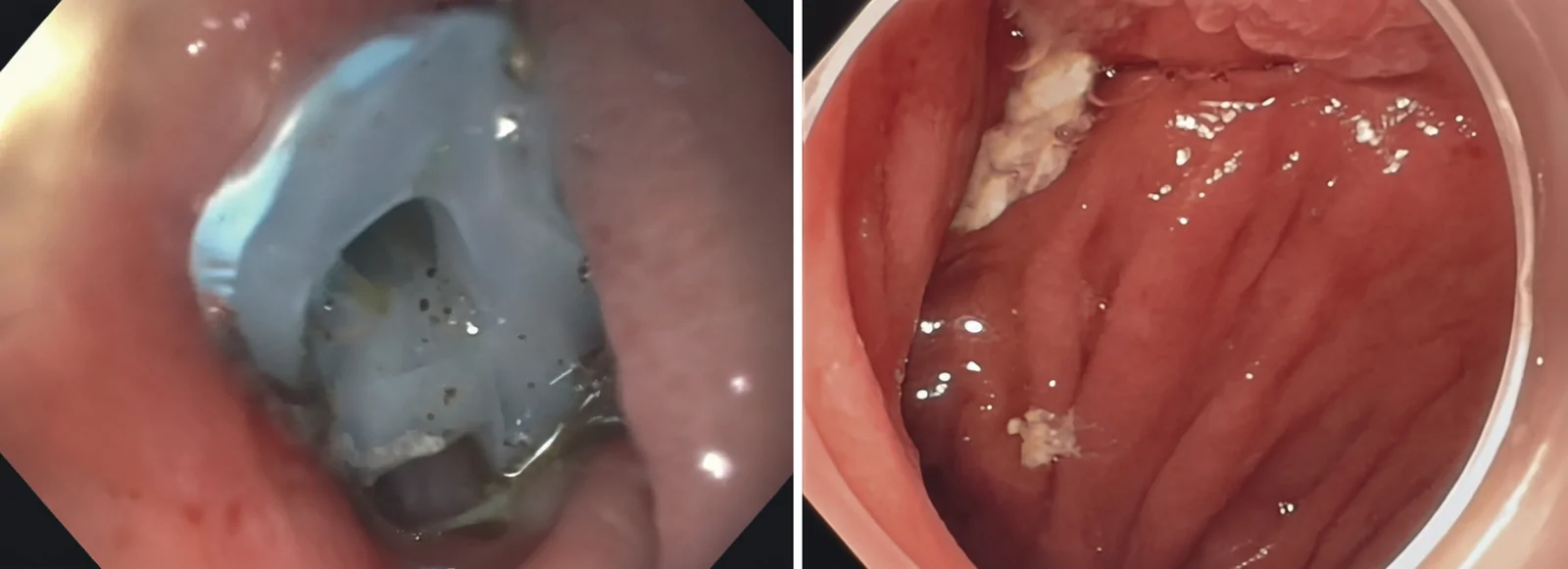
Pre- and post-treatment endoscopic images. Left: anastomotic dehiscence with visualization of the surgical abdominal drainage through the wall defect. Right: complete resolution of the dehiscence after treatment.

This case highlights the feasibility and effectiveness of combining duodenal exclusion with trans-mesh biliary and pancreatic stenting as a minimally invasive alternative to high-risk surgical re-intervention for complex duodeno-jejunal leaks.

Endoscopy_UCTN_Code_CPL_1AH_2AG

## Declaration of GenAI Use

The authors confirm that no generative artificial intelligence was used in the preparation of this manuscript.
